# Fat and exposure to 4-nitroquinoline-1-oxide causes histologic and inflammatory changes in murine livers

**DOI:** 10.1371/journal.pone.0268891

**Published:** 2022-05-31

**Authors:** Lenore D. Pitstick, Joanna Goral, Ryan A. Schmelter, Christine M. Fuja, Mae J. Ciancio, Matthew Pytynia, Alice Meyer, Jacalyn M. Green

**Affiliations:** 1 Department of Biochemistry and Molecular Genetics, College of Graduate Studies, Midwestern University, Downers Grove, IL, United States of America; 2 Department of Anatomy, College of Graduate Studies, Midwestern University, Downers Grove, IL, United States of America; 3 Chicago College of Osteopathic Medicine, Midwestern University, Downers Grove, IL, United States of America; 4 Department of Biomedical Sciences, College of Graduate Studies, Midwestern University, Downers Grove, IL, United States of America; Texas A&M University, UNITED STATES

## Abstract

Risk factors for liver cancer include tobacco use, alcohol consumption, obesity, and male sex. Administration of 4-nitroquinonline-1-oxide (4NQO) in drinking water mimics the effects of tobacco and leads to oral carcinoma in mice. This study compared the effects of diets high and low in saturated fat (HF and LF, respectively), and sex, on liver histopathology in 4NQO-treated mice and controls. We hypothesized that 4NQO would cause histopathological changes in liver, and that a HF diet would increase hepatic pathology when compared to the LF diet. Mice (C57Bl/6, 36/sex), were divided into a low fat (10 kcal% fat; LF) or high fat (60 kcal% fat, HF) diet. Mice were further subdivided into one of 3 water treatment groups for 17 weeks: water (control), vehicle (1.25% propylene glycol in water [PG]), or 4NQO in (50 μg/ml; 4NQO). All mice were subsequently given water alone for 6 more weeks. Upon euthanasia, livers were harvested, fixed, sectioned, and stained with hematoxylin and eosin (H&E). H&E slides were graded for histopathology; frozen liver samples were analyzed for triglyceride content. Trichrome stained sections were graded for fibrosis. CD3+ T cells, CD68+ macrophages, and Ly6+ neutrophils were detected by immunohistochemistry. Compared to water controls, 4NQO-treatment caused mouse liver histopathological changes such as fibrosis, and increases in hepatic neutrophils, T cells, and macrophages. HF diet exacerbated pathological changes compared to LF diet. Male controls, but not females, demonstrated severe steatosis and increased triglyceride content. 4NQO treatment decreased hepatic fat accumulation, even in animals on a HF diet. In conclusion, this murine model of oral cancer may serve as a model to study the effects of tobacco and diet on liver.

## Introduction

Globally, liver cancer ranks fourth in cancer mortality, while constituting the sixth most common cancer [1]. In general, men are 2 to 3 times more likely to be diagnosed with liver cancer than women; mortality rates likewise mirror this difference. About ¾ of liver cancers are hepatocellular carcinoma (HCC) [2]. The rates of HCC vary in different parts of the world, primarily because the rates of contributory risk factors vary as well [3, 4]. Risk factors include infection with hepatitis B or C virus, alcohol abuse disorder, diabetes mellitus, alcoholic and non-alcoholic fatty liver disease, obesity, and tobacco use [2, 5].

In the United States, between 2003 and 2012, while mortality rates for most cancers declined, the incidence and mortality rates of liver cancer increased for both men and women [6, 7]. These increases are attributed largely to the observation that “Baby Boomers”, defined as those born between 1945–1965, have rates of hepatitis C infection 6-times greater than other American adults [8]. It is also increasingly being recognized that growing rates of overweight/obesity [9, 10] and metabolic syndrome [11] likely contribute to increased HCC risk. An analysis of individual risk factors for HCC using the Surveillance, Epidemiology, and End Results Medicare database demonstrated that the highest individual population attribution fraction for HCC was diabetes mellitus/obesity [12]. Diet, activity levels, and related lifestyle factors therefore impact risk for liver cancer.

Animal models facilitate the identification of mechanisms of disease development and progression, as well as enable testing of candidate treatments. There are many murine models for both alcoholic and non-alcoholic fatty liver disease, including those progressing to steatohepatitis and HCC [13–19]. These models typically utilize C57Bl/6 male mice due to their propensity to gain body weight and develop fatty livers, as well as progress to more advanced disease [16]. Diets vary in macronutrient composition, but are often high in fat, although the type of fat may vary. Some models incorporate choline deficiency or a toxic agent, including carbon tetrachloride (CCl_4_) [20], diethylnitrosamine [21, 22], or streptozotocin [23], to accelerate hepatocellular changes. These chemicals accelerate cancer development against a backdrop of dietary manipulation, a so-called ‘two-hit’ mechanism of liver damage.

Smoking is a major risk factor for about 30 percent of all human cancers, including liver cancer [24, 25]; tobacco use also increases the mortality for liver cancer [26]. It is difficult, however, to accurately duplicate the effects of smoking in an animal model. The water-soluble carcinogen 4-nitroquinoline-1-oxide (4NQO) causes formation of DNA adducts, altered repair of DNA, and oxidative stress, causing molecular changes similar to those caused by tobacco use [27]. Administration of 4NQO to mice through addition to their drinking water causes oral cancer at rates approaching 100% and thus is an established model of oral squamous cell carcinoma [28–30].

In this study we investigated the effects of sex, low fat versus high fat diet, and administration of 4NQO on pathological changes in livers of C57Bl/6 mice. Hepatic T cells, macrophages and neutrophils were quantified to characterize the involvement of different lymphoid cell populations in immune response to 4NQO treatment, and to identify whether these populations were affected by dietary fat content. A major goal was to identify if 4NQO administration to mice might prove to be a useful model for study of liver pathology.

## Materials and methods

### Animal model

Our studies followed the University’s guidelines for the safe and humane use of experimental animals as required by Midwestern University’s Institutional Animal Care and Use Committee, which approved all animal procedures (protocol #2501, Downers Grove, IL). Male and female C57Bl/6 mice (36/sex; 5 weeks old; Jackson Labs) were randomly assigned to either a low-fat diet (LF, 10 kcal% saturated fat, Research Diets, New Brunswick, NJ) or a high-fat diet (HF, 60 kcal% saturated fat; Research Diets). Detailed compositions of the diets are available in S1 and S2 Tables. Then, mice were further subdivided into one of three water treatment groups: water control (n = 3/sex/diet; H_2_O); propylene glycol water control (1.25% propylene glycol [PG] in water; n = 5/sex/diet; Sigma, St. Louis, MO); and 4NQO in PG-H_2_O (50 μg/mL; 4NQO, n = 10/sex/diet; Sigma, St. Louis, MO). PG-H_2_O and 4NQO administration were stopped after 17 weeks, after which all mice were provided water alone for 6 more weeks. The experimental timeline of the protocol is pictured in S1 Fig. Each animal’s behaviors and appearance were inspected daily according to guidelines of the NIH Office of Laboratory Welfare for humane endpoints before the end of the study. Accordingly, if mice appeared distressed, failed to eat, or lost 20% of their body weight within a week, they were euthanized. Body weights and tumor burden were recorded weekly. Twenty-four-hour food intake was measured during week 19 of the experimental study to ensure that food intake was not compromised by the tumor burden. At the end of the protocol at week 23, mice were euthanized with carbon dioxide for five minutes, followed by cervical dislocation. Then complete autopsies were performed. Mice were weighed at the beginning of the dietary treatment and weekly thereafter. At sacrifice livers were weighed, and portions were flash frozen and other portions were fixed in formalin (Sigma-Aldrich, St. Louis, MO), embedded in paraffin, and sectioned at 7 μm. Tissue embedding was performed using a Tissue-Tek TEC Embedding Station and a Thermo Scientific MICROM HM 325 microtome; both instruments were in the Midwestern University Core Facility, Downers Grove, IL.

### Histology evaluation

Liver sections were stained with hematoxylin and eosin (H&E, Sigma-Aldrich, St. Louis, MO) and examined microscopically by at least three independent researchers. To evaluate liver histopathology we used grading adapted from the liver pathology literature [31]. We evaluated liver tissue architecture for the loss of regular arrangement of hepatocytes, for variability in cell and nuclear size, for the presence of inflammation as evidenced by periportal and intralobular infiltrates of lymphoid cells, and fibrosis. The presence of preneoplastic lesions, characterized by areas of high cellular density and an increase in nuclear-to-cytoplasmic ratio in hepatocytes, was also recorded. Steatosis on H&E slides was analyzed on a 0 to 3 scale. A score of 0 represented no fatty change in the tissue section; a score of 1 represented only mild fatty changes (<25% of the area); a score of 2 represented fatty changes between 25–50% of the area; and a score of 3 represented fatty changes >50% of the area. Fibrosis was graded using tissue sections stained with trichrome, which visualizes connective tissue. Manufacturer’s instructions were followed for Trichrome Stain (ab150686, Abcam, Cambridge, MA), except that incubation with Bouin’s solution was done overnight at room temperature instead of 60°C for one hour. Compared to humans, murine hepatic connective tissue is less abundant and fibrotic changes are less distinct [32]. Importantly, both fibrous bridges between periportal and pericentral regions and cirrhosis do not develop in the mouse liver. Trichrome-stained slides were graded using a fibrosis scale, ranging from 0 to 3. A score of 0 represented no fibrosis and was characteristic of normal hepatic tissue. A score of 1 represented a small amount of connective tissue visible primarily in larger portal triads. A score of 2 was assigned when connective tissue was present in both small and large portal triads. A score of 3 included an expansion of fibrosis whereby connective tissue was also present in liver parenchyma between hepatocytes.

### Triglyceride measurement

Samples of frozen liver were hydrolyzed into free fatty acids and glycerol; the latter was measured using a colorimetric test as an indicator of triglyceride levels (Bio-protocol, Vol 2, ISS/3, July 05, 2012). Liver tissue samples (100–300 mg) were incubated with ethanolic KOH (2 parts ethanol: 1 part 30% KOH) overnight at 55°C. After addition of 1M MgCl_2_ and centrifugation, glycerol levels of the supernatant were calculated from the absorbance at 540 nm, using a coupled enzyme reagent (Free Glycerol Reagent, F64428, Sigma-Aldrich, St. Louis, MO). Readings were determined by comparison to a glycerol standard curve, using glycerol concentrations between 0 and 1 mg/mL. Samples were done in duplicate at various dilutions and normalized to the weight of the liver.

### Alanine Aminotransferase (ALT)

Serum was stored at -80°C and ALT was measured using a kit (ab105134, Abcam, Cambridge, MA) and a Perkin Elmer EnSpire Plate Reader located in the Midwestern University Core Facility, Downers Grove, IL.

### Immunohistochemistry

Standard immunohistochemical (IHC) techniques were employed to detect T lymphocytes (CD3+), macrophages (CD68+), and neutrophils (Ly6+). Serial tissue sections were deparaffinized in 100% Micro-Clear (Micron Environmental Industries, Alexandria, VA) and rehydrated in decreasing concentrations of alcohol. Antigen retrieval was performed in citrate buffer, pH 6.0 (Sigma-Aldrich, St Louis, MO) at 96°C, for 15 min. Endogenous peroxidase was quenched by 10 min incubation with Bloxall (SP-6000, Vector Laboratories, Burlingame, CA). Nonspecific binding was blocked by 1-hr incubation with 10% horse or goat serum/1% BSA/Tris buffered saline (TBS) blocking solution, depending on the primary/secondary antibody combination. Slides were then incubated overnight at 4°C, with one of the following primary antibodies: rabbit anti-CD3 (1:500, ab16669, lot number GR320743-1, Abcam, Cambridge, MA); rabbit anti-CD68 (1:2000, ab125212, lot number GR77836-39, Abcam); or rat anti-Ly6 (1:500, ab2557, lot number GR277603-1, Abcam). Primary antibodies were diluted in 2.5% horse or goat serum/TBS. Specificity of the antibodies was confirmed by performing IHC on spleen tissue sections [33]. After TBS washes, the slides were incubated for 30 min with the appropriate secondary antibody horseradish peroxidase (HRP) conjugate (horse anti-rabbit IgG [MP6401] or goat anti-rat IgG [MP7444] ImmPRESS HRP Reagent kits [Vector Laboratories, Burlingame, CA]). This was followed by a 2 min incubation with 3,3’-Diaminobenzidine (DAB) HRP substrate (Vector Laboratories). Finally, tissue sections were counterstained with Mayer’s hematoxylin for 10 seconds. Primary and secondary antibody control studies were also performed. Positively stained cells were counted in three different fields at 400x magnification, averaged, and normalized to the area of the field viewed (0.196 mm^2^).

### Statistical analysis

Data from control mice that were provided water with and without PG were compared using the Student’s T-test, and if they were not significantly different, these groups were combined as a single control group for further analysis. A three-way ANOVA was used to measure significance in triglyceride levels, as well as to examine differences in numbers of T lymphocytes (CD3+), macrophages (CD68+), and neutrophils (Ly6+). A post-hoc Bonferroni test for multiple comparisons was subsequently performed. Ordinal values, including the steatosis and fibrosis grades, were analyzed using the Kruskal-Wallis analysis, with a Dunn’s post-hoc test for multiple comparisons. Data were analyzed using GraphPad Prism Version 9.0 for Windows (GraphPad Software, San Diego, CA). Data are displayed as mean or median ± standard deviation (SD).

## Results

In this study we aimed to investigate whether administration of the chemical carcinogen 4NQO would cause hepatic pathology in an established mouse model of oral cancer. 4NQO, when administered in the drinking water, caused oral squamous cell carcinoma in all mice that completed the protocol [34]. This chemical carcinogen causes changes in oral tissues in a manner analogous to changes caused by tobacco exposure. Of the 72 animals in the study, three animals were euthanized before the end of the protocol, following guidelines of NIH Office of Laboratory Welfare in the guide for the Institutional Animal Care and Use Committees for humane endpoints. These mice had hunched backs, ruffled fur and were lethargic. The first animal (HF 4NQO female) was euthanized 25 days before the end of the study; no tumors were detected in this mouse. The second animal (HF 4NQO female) was euthanized 5 days before the end of the study; she developed two large oral tumors (4.5 mm and 5.5 mm in size). The third animal (HF 4NQO male) was sacrificed 15 days before the end of the study; this mouse developed a large 1 cm tumor that was located under the tongue. This tumor was the largest noted in the study and weighted 0.32g, which constituted approximately 1% of the body weight of this mouse. The Office of Laboratory Welfare defines tumor weight exceeding 10% of normal body weight as the indication for the humane endpoint. Therefore, none of the experimental animals developed tumors that, due to their size, would mandate euthanizing the animal. This was further confirmed by the 24-hour food intake measurement performed at week 19 of the study that did not show any significant reduction in calorie consumption in 4NQO-treated when compared to control mice [34].

Since liver cancer risk is increased in smokers, and liver pathology increases in humans with a high fat diet, we also compared effects of a HF diet versus a LF diet in males and females. Gross visual inspection of livers collected upon euthanasia revealed no obvious lesions. However, in HF mice, all male control livers were pale when compared to female control livers and to livers from mice exposed to 4NQO. Liver weights were compared, and the livers of the control males fed a HF diet weighed significantly more (2.99 g ± 0.82) than the livers from all other treatment groups (0.90 g to 1.66 g, Table 1). When the liver weight was presented as a percentage of the body weight (i.e., as the liver index), the differences among the groups decreased, although the HF male controls still had the highest liver index.

**Table 1:** Comparison of liver weight and liver index among treatment groups.

Treatment Group	Liver weight (g)	Mouse weight (g)	Liver index (liver weight/body weight) %
Female, LF, Control	1.15 ± 0.08	22.5 ± 0.94	5.10 ± 0.26
Female, HF, Control	1.24 ± 0.11	38.1 ± 4.9	3.25 ± 0.29
Male, LF, Control	1.66 ± 0.23	36.9 ± 3.8	4.50 ± 0.34
Male, HF, Control	2.99 ± 0.82	52.0 ± 3.4	5.75 ± 1.2
Female, LF, 4NQO	0.94 + 0.09	18.9 ± 1.6	4.97 + 0.28
Female, HF, 4NQO	0.86 + 0.13	20.4 ± 3.1	4.22 + 0.38
Male, LF, 4NQO	1.02 ± 0.18	23.8 ± 3.0	4.28 ± 0.54
Male, HF, 4NQO	0.90 ± 0.27	24.0 ± 6.3	3.75 ± 0.56

### Histology evaluation

**Fig 1:**
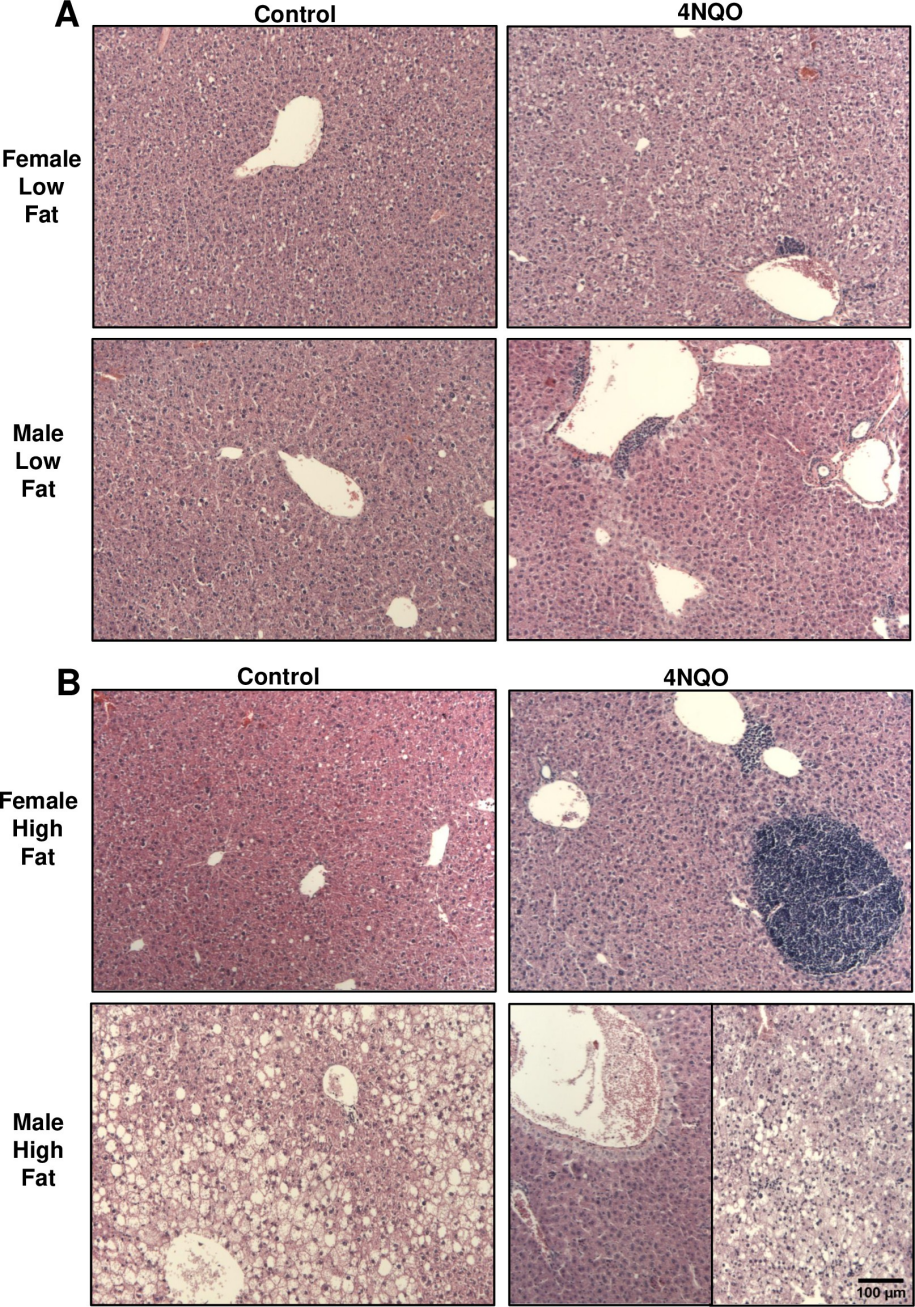
Images of H&E-stained tissue sections from representative livers from the indicated treatment group (100x magnification). (A) Control and 4NQO-treated male and female mice on a LF diet. (B) Control and 4NQO-treated male and female mice on a HF diet.

Examination of the H&E-stained hepatic tissues showed that males and females on the LF diet (controls) displayed normal liver architecture. Male and female mice treated with 4NQO and on a LF diet displayed accumulations of lymphoid cells indicating hepatitis (Fig 1A). Male mice on HF diet and exposed to 4NQO demonstrated the highest degree of liver histopathological changes among all treatment groups (Fig 1B). Of the ten mice in this group (4NQO-treated males on HF diet), seven mice showed preneoplastic lesions and aggregates of lymphoid cells in periportal areas and liver parenchyma as well as absence of steatosis. Three of those seven mice demonstrated additional presence of necrotic foci with eosinophilic cells and scattered balloon cells. The remaining three 4NQO-treated males on HF diet displayed a very different phenotype, with prevalent microvesicular steatosis equally present in zones 1 to 3 (Fig 1B). Multiple aggregates of lymphoid cells were also present, located in the parenchyma, periportal and pericentral areas. All female mice on the HF diet and treated with 4NQO showed the presence of lymphoid cell aggregates and lack of fatty change. The C57Bl/6 control males (i.e., those not treated with 4NQO) were significantly more affected by the HF diet than the control females on the same diet, as shown by the accumulation of lipid in their hepatocytes (Fig 1B). When present in control mice, steatosis was largely macrovesicular, and primarily associated with pericentral hepatocytes (Zone 3), often extending into Zone 2 and occasionally into periportal Zone 1 (Fig 1B). Females did not develop steatosis, regardless of their diet and treatment with 4NQO (Fig 1).

### Steatosis

**Fig 2:**
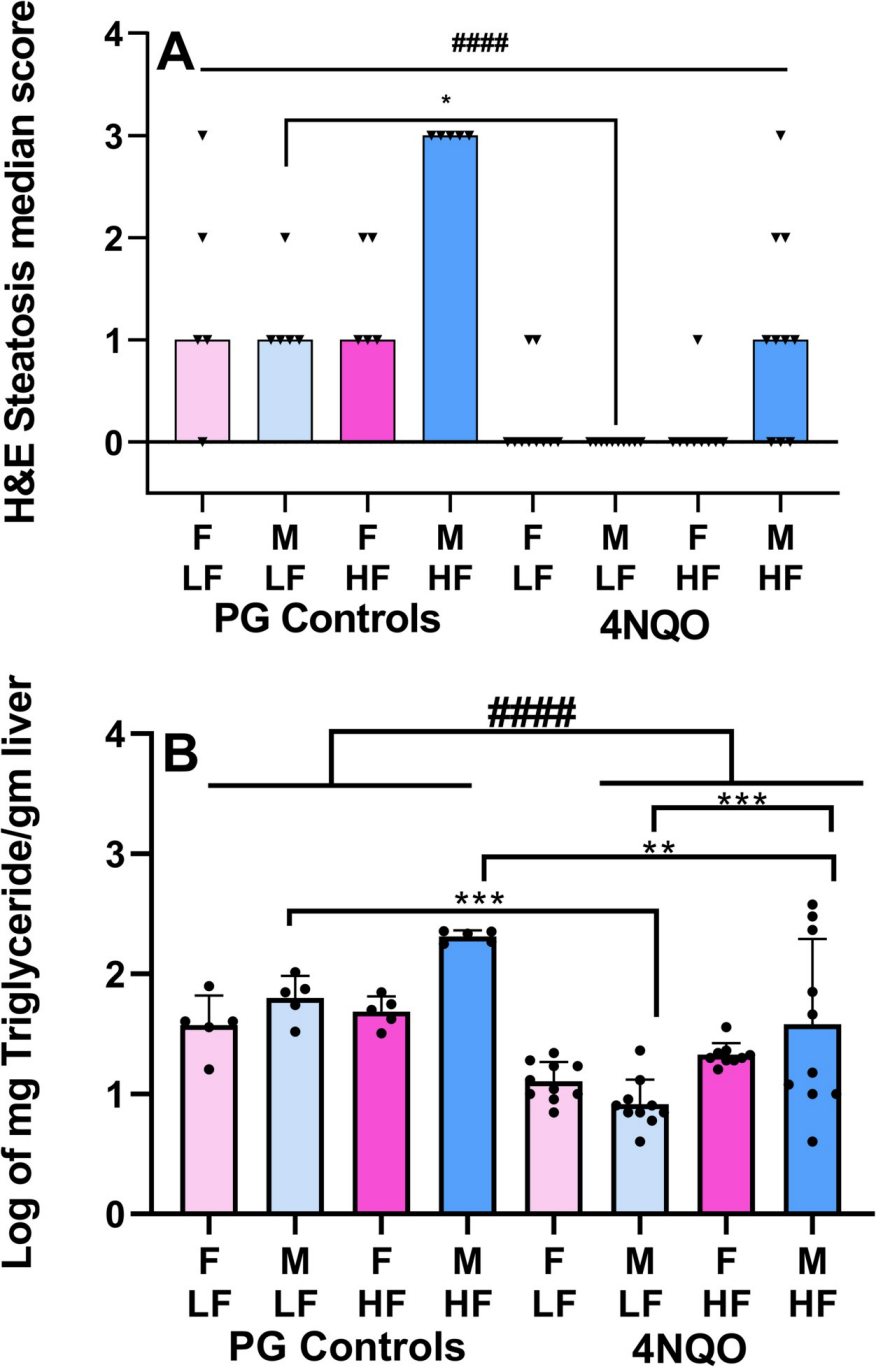
Hepatic fat content. (A) Hepatic steatosis score from histological assessment of mice from all treatment groups. Data shown are median values. #### refers to the Kruskal-Wallis statistical analysis. * refers to the Dunn’s post-hoc test for multiple comparisons. (B) Log of triglyceride levels in livers of mice from all treatment groups. Data shown are mean values ±SD. #### refers to the 3-way ANOVA. * refers to results from the Bonferroni post-hoc test for multiple comparisons.

Liver fat content was assessed by microscopic semi-quantitative analysis of H&E-stained liver sections and by measurement of hepatic triglycerides. First, each H&E-stained tissue section was graded on a scale of a 0 to 3. The Kruskal-Wallis statistical analysis confirmed that there was a significant difference in the medians (p< 0.0001). The subsequent Dunn’s multiple comparison test demonstrated that steatosis scores of livers from 4NQO-treated males fed a LF diet were significantly lower than scores from LF male controls (p = 0.0431). When comparing scores from control mice that were not treated with 4NQO, livers from control males on a HF diet displayed the highest steatosis scores (Fig 2A). Treatment with 4NQO caused substantial decreases in steatosis scores. Measurement of hepatic triglyceride content using a colorimetric test corroborated the observations made from assessing the steatosis in the H&E-stained tissue sections (Figs 2B and S2). The 3-way ANOVA performed on the log of the triglyceride measurements showed that all three variables demonstrated significance: 4NQO treatment (Fig 2B, p < 0.0001), sex (S2A Fig, p = 0.016) and diet (S2B Fig, p = 0.0001) and that sex interacted with diet (S2 Fig, p = 0.026). As shown in Fig 2B, a post-hoc Bonferroni test for multiple comparisons demonstrated significantly higher triglyceride levels in: LF control males compared to LF 4NQO-treated males (p = 0.0002); HF male controls compared to HF males treated with 4NQO (p = 0.0024); 4NQO-treated males on a HF diet compared to 4NQO-treated males on a LF diet (p = 0.0006). HF control male livers contained the highest amount of triglyceride, 205.8 ± 23.5 mg/g liver. In contrast, 4NQO-treated males on a HF diet displayed great variation in fat content; with a mean of 107.7 ± 141 mg/g liver, hepatic triglyceride levels ranged from 4 to 377 mg/g liver. This correlated with the histological presentation of liver tissues (Fig 1).

### Fibrosis

**Fig 3:**
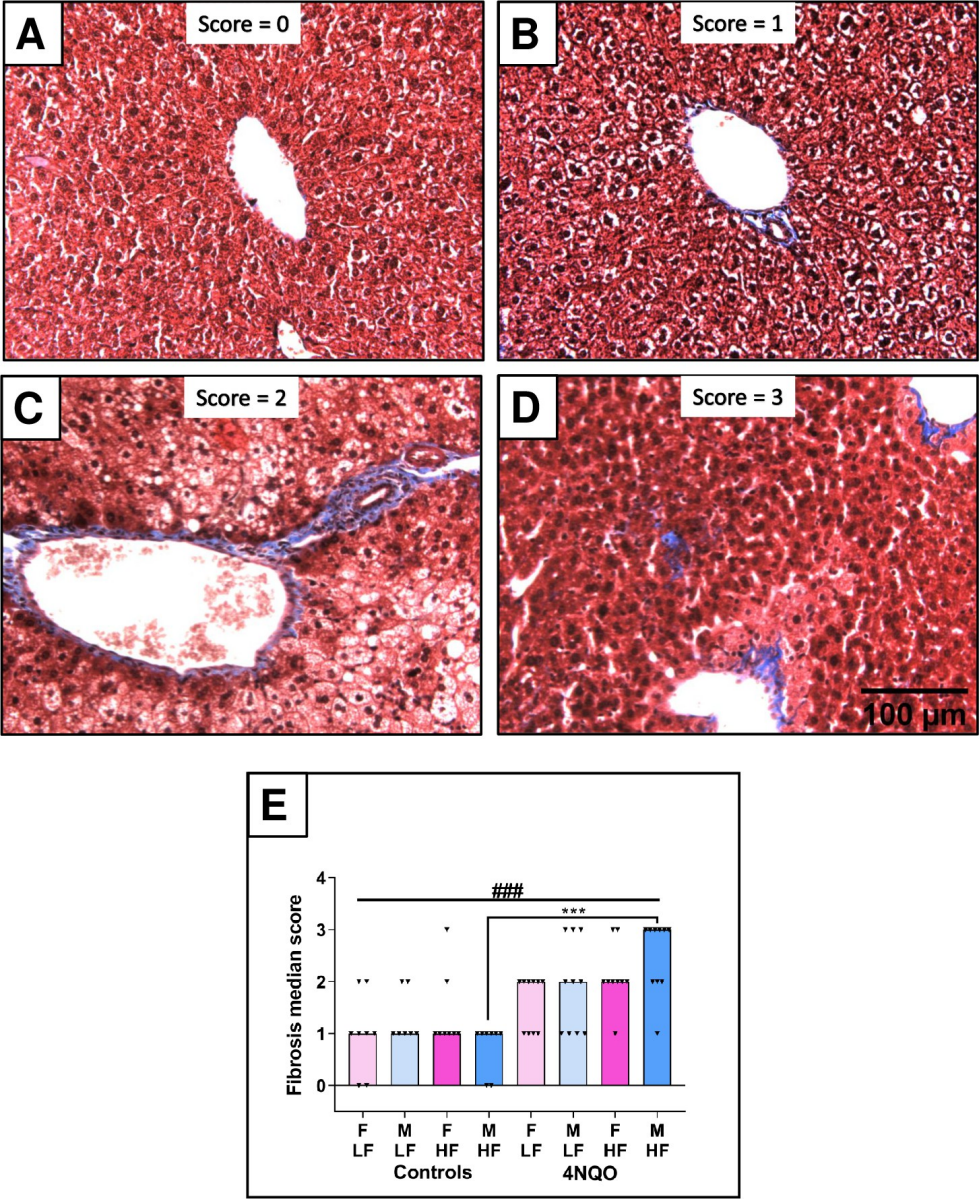
Assessment of fibrosis in trichrome-stained representative liver tissue section (200X). (A) Fibrosis grade of 0. Tissue shown is from a LF control female. (B) Fibrosis grade of 1. Tissue shown is from a LF 4NQO-treated female. (C) Fibrosis grade of 2. Tissue shown is from a LF 4NQO-treated female. (D) Fibrosis grade of 3. Tissue shown is from a HF 4NQO-treated male. (E) Graph of the median fibrosis score of each treatment group. ### refers to the Kruskal-Wallis statistical analysis. *** refers to the Dunn’s post-hoc test for multiple comparisons.

Hepatic fibrosis was assessed by semi-quantitative analysis using trichrome stained liver tissue sections (Figs 3 and S3). Dietary fat content had no effect on amount of connective tissue in control male and female mice (median fibrosis score of 1). The Kruskal-Wallis statistical analysis demonstrated significant differences of the medians (p = 0.0001). The Dunn’s post-hoc test for multiple comparisons showed that fibrosis was higher in 4NQO-treated males on a HF diet compared to control males on a HF diet (p = 0.0002). When present, fibrosis was relatively mild, and primarily associated with periportal zone 1 (Figs 3B and 3C and S3). Livers from male mice on the HF diet and exposed to 4NQO showed the most fibrotic changes and were the only group with collagen deposits in liver parenchyma.

### Lymphoid cell analysis

**Fig 4:**
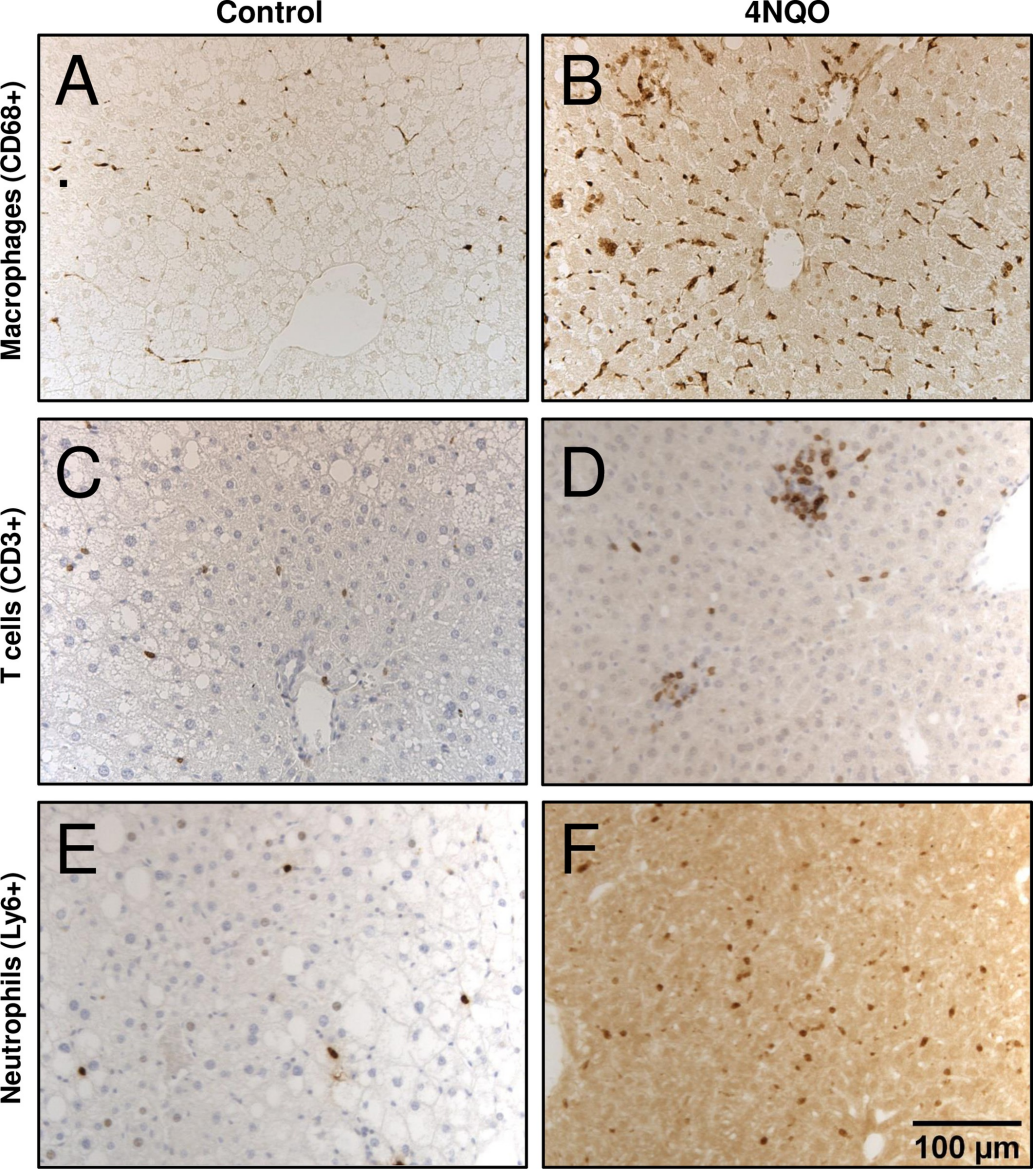
Lymphoid cell infiltration in representative liver sections from control mice or mice treated with 4NQO (200x). All hepatic samples are from male mice on a HF diet. (A) CD68+ macrophages in a control liver (B) CD68+ macrophages in a 4NQO-treated liver (C) CD3+ T cells in a control liver (D) CD3+ T cells in a 4NQO-treated liver. (E) Ly6+ neutrophils in a control liver (F) Ly6+ T cells from a 4NQO-treated liver.

**Fig 5:**
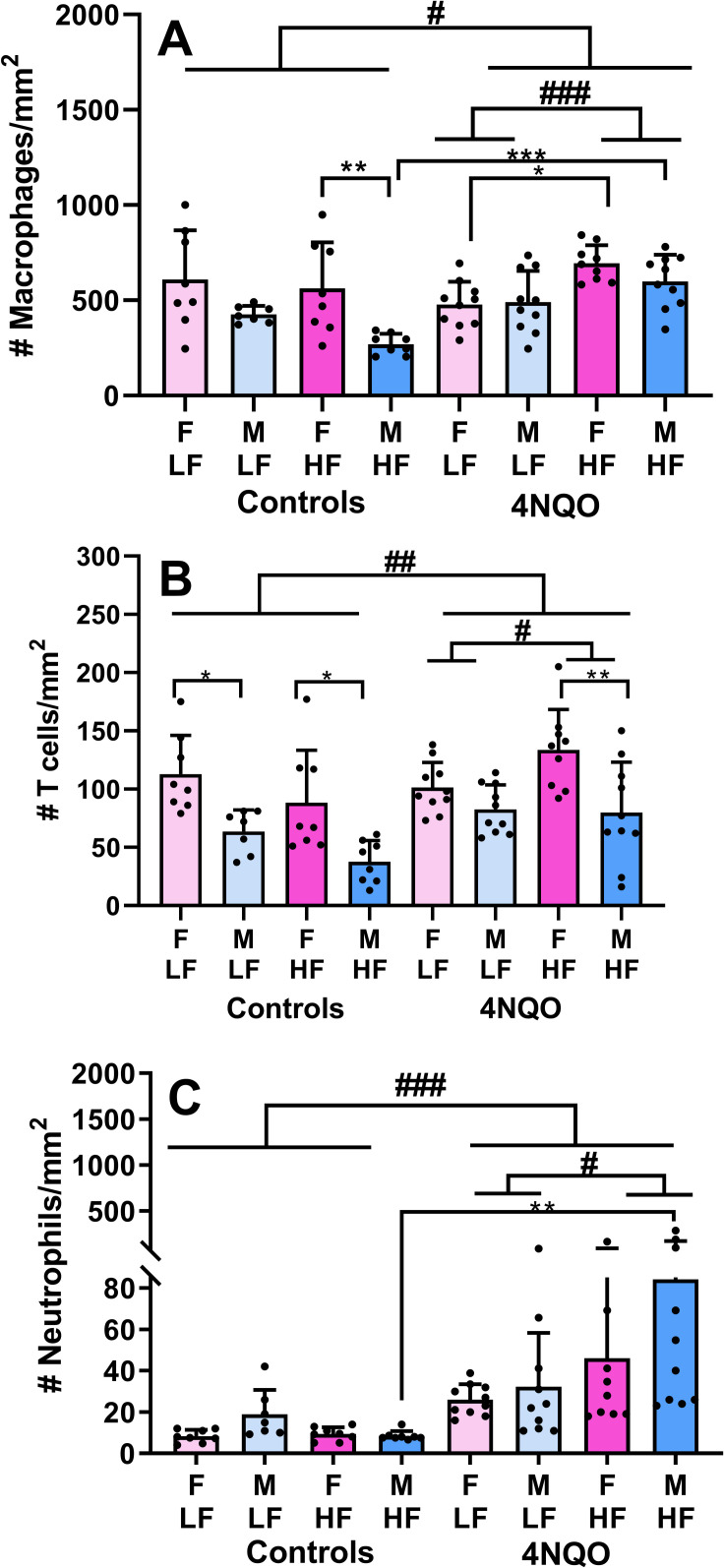
Hepatic lymphoid cell counts from each treatment group. (A) CD68^+^ macrophages per mm^2^. (B) CD3+ T cells per mm^2^. (C) Ly6+ neutrophils per mm^2^. Data shown are mean values ± SD. # refers to the 3-way ANOVA. * refers to results from the Bonferroni post-hoc test for multiple comparisons.

The presence of macrophages, T lymphocytes, and neutrophils in livers was investigated using immunohistochemical staining for CD68, CD3, and Ly6, respectively (Figs 4 and 5 and S4–S8). Macrophages were the most abundant inflammatory cell type in liver sections and were primarily associated with sinusoids. T lymphocytes were scattered in liver parenchyma and constituted the dominant cell type within lymphoid cell aggregates. Neutrophils were present in liver parenchyma; they were not present in lymphoid cell aggregates.

Quantification of lymphoid cells were analyzed first with a 3-way ANOVA, followed by a Bonferonni post-hoc test for multiple comparisons. Treatment with 4NQO caused a 20% increase in hepatic macrophages; 4NQO-treated livers and control livers contained 562±155 and 468±221 macrophages/mm^2^, respectively (Fig 5A, p = 0.0116). Similarly, 4NQO treatment was associated with a 29% increase in hepatic T cells; 4NQO-treated livers and control livers had 98.5 ± 36.9 and 76.0 ±41.3 T cells/mm^2^, respectively (Fig 5B, p = 0.0026). The greatest increase was observed in neutrophils, which increased 400% in livers from mice treated with 4NQO. 4NQO-treated livers and control livers contained 47.1 ± 54.9 and 11.0 ± 7.2 neutrophils/mm^2^, respectively (Fig 5C, p = 0.0003). Thus, macrophages, the most common hepatic lymphoid cell type, increased the least in mice treated with 4NQO. The increase in lymphoid cells associated with 4NQO treatment was affected by diet whereby mice on a HF diet had higher numbers of macrophages (p = 0.0008, Fig 5A), T cells (p = 0.0102, Fig 5B), and neutrophils (p = 0.0354, Fig 5C) compared to their LF counterparts. The 3-way ANOVA also showed that among 4NQO-treated mice, females had higher levels of hepatic macrophages than males (p = 0.0114, S7A Fig). Moreover, females overall had higher numbers of macrophages than males (p = 0.0005, S7B Fig). Similarly, females had higher numbers of T cells than males (p<0.00001, S8 Fig).

The Bonferroni post-hoc test for multiple comparisons revealed some statistically significant and interesting results. For macrophages, among controls, HF females had higher macrophage counts than HF males (Fig 5A, p = 0.005). Among HF males, treatment with 4NQO resulted in higher numbers of macrophages than in controls (p = 0.0005). Among 4NQO-treated females, those on a HF diet had higher numbers of macrophages than those on a LF diet (p = 0.0442). For T cells, among controls, LF females had more T cells than LF males (Fig 5B, p = 0.043) and HF females had more T cells than HF males (p = 0.024). Similarly, 4NQO-treated HF females had higher numbers of T cells than 4NQO-treated HF males (p = 0.005). Finally, HF 4NQO-treated males had higher numbers of neutrophils than HF control males (Fig 5C, p = 0.0017).

### Serum ALT

All tests for ALT fell within the reference range for the kit (S9 Fig).

## Discussion

While this study investigated the effects of 4NQO on livers in male and female mice on a HF diet or LF diet, a previous study focused upon the tongues from the same animals [34]. All 4NQO-treated mice developed tumors in the oral cavity, which is not surprising given that this is an established model of oral cancer. A novel finding, however, was that the final tumor burden was significantly greater in mice on a HF diet then in those on the LF diet, and the former group also had a higher lingual histopathology score. Mice with oral cancers, regardless of diet, displayed cachexia that correlated with loss of lingual fat content. Interestingly, a 24-hour analysis of food intake showed that calorie consumption was not significantly different between 4NQO-treated mice and corresponding controls.

Our current results demonstrate that, in the timeline of this study, 4NQO-treatment did not result in liver cancer; however, we did observe increased liver pathology and a possible presence of precancerous lesions as well as development of hepatitis with administration of 4NQO and a HF diet. In control mice (not treated with 4NQO) the HF diet resulted in significantly increased hepatic triglyceride content, which was far more appreciable in males than females; this is consistent with the literature on C57Bl/6 mice [35]. Treatment with 4NQO did reduce the general level of steatosis in the livers; however, HF diet 4NQO-treated males displayed two distinctive phenotypes. Livers of one group showed high fat content and steatosis, while livers of the other group were characterized by high cell density with small hepatocytes displaying high nuclear-cytoplasmic ratio (Fig 1B).

In this study, oral exposure to 4NQO over a period of about 4 months resulted in hepatic fibrosis (Fig 3) as well as increased lymphoid cell infiltration (Figs 4 and 5). Since shortly after its synthesis in the early 1950’s [36], 4NQO has been used as a carcinogen in rodents. 4NQO delivery has involved a range of methods, including direct application to skin, subcutaneous or intraperitoneal injection, or addition to drinking water. Early work utilized radio-labeled 4NQO delivered orally to rats via a stomach tube, to identify what tissues accumulated the compound as well as to identify modes of metabolism and elimination from the body [37]. Radioactive 4NQO was highest in the liver an hour after the dose, and decreased over time thereafter, as the body metabolized the compound. Most activity, however, was associated with gastrointestinal tissue or its contents. In a similar experiment, ^3^H-labeled 4NQO was injected subcutaneously into mice [38]. Radioactivity was monitored in liver, lung, spleen, and blood. The small amount of radioactivity taken up by the liver dropped substantially within 24 hours. In contrast, lungs exhibited almost ten times more label, which likewise dropped within 24 hours. Chemical analysis demonstrated that the metabolites retained by the liver were largely benign. Intraperitoneal and subcutaneous injection of 4NQO into newborn rats was used to detect DNA damage in various tissues [39]. While DNA damage was measured in the liver, histological analysis detected no evidence of hepatic cancer. Later, researchers measured the mutation frequencies in various organs following a single dose of 4NQO administered either by gavage or intraperitoneally [40]; they found a high mutation frequency in the liver, using a dose of 200 mg/kg by gavage. They concluded that the mutagenic potential of 4NQO depended on the mode of delivery. Our results are consistent with these reports. While no liver cancer was observed, hepatitis and preneoplastic lesions were noted, which demonstrated that 4NQO and its metabolites did have adverse effects on hepatic tissue.

In the presence of 4NQO, the HF diet was associated with significantly increased hepatic cell counts of macrophages, T cells, and neutrophils (Fig 5). Interestingly, in the absence of 4NQO, diet did not affect lymphoid cell infiltration of liver. The most abundant hepatic lymphoid cells we measured were the macrophages. This was not unexpected, since Kupffer cells normally are the most numerous lymphoid cell type resident in the liver [41]. Another interesting observation, however, was that females had higher levels of hepatic lymphoid cell infiltration (macrophages and T cells) when compared to their male counterparts, in both 4NQO-treated mice as well as controls (Figs 5 and S7B and S8). In our study neutrophils showed the highest increase in 4NQO-treated mice indicating development of inflammation. Uehara et al describe a murine model of hepatocellular carcinoma that consists of a single initial injection of N-nitrosodiethylamine followed by periodic injections of carbon tetrachloride [42]. This protocol results in all mice developing liver cancer by 5 months of age, and unlike many murine models, includes both hepatic inflammation and increased fibrosis. While we did not observe obvious hepatocellular carcinoma, we did observe increased cell density and fibrosis with 4NQO treatment, as well as increased lymphoid cell infiltration that was augmented by a HF diet. More recently, a murine model has been developed that involves treatment with carbon tetrachloride in combination with a Western diet, which was defined as high-fat, high-fructose, and high cholesterol [20]. In this model, steatosis, fibrosis, and inflammation were observed that resembled patterns of liver injury found in humans with non-alcoholic steatohepatitis. The combination of the carcinogen and the Western diet resulted in hepatocarcinoma development in 6 months. We too found that the combination of carcinogen exposure and a diet, in our case 4NQO and HF diet, amplified the effects of each.

In summary, we investigated the possibility that administration of the carcinogen 4NQO in drinking water, an established model of oral cancer that induces tissue changes similar to tobacco use, might also result in pathology in liver. The sample size of n = 70 mice for the study had adequate power to detect effect sizes for the overall effects of 4NQO treatment (effect size = 0.20, power = 70%), diet (effect size = 0.25, power = 86.90%), and sex (effect size = 0.26, power = 90.25%) as well as the interaction between diet and sex (effect size = 0.21, power = 73.15%) at alpha = 0.05 using a three-way ANOVA for the triglyceride data. We found that 4NQO did cause increased histopathology and fibrosis, as well as increased lymphoid cell infiltration, and that the HF diet heightened some of these effects. Moreover, treatment with 4NQO largely abolished hepatic fat accumulation in mice on a HF diet. It is possible that a longer period of exposure to 4NQO, or a more direct mode of administration, might have resulted in more dramatic changes. Since smoking and HF diet contribute to increased risk of many cancers, including liver, this model may prove useful for future investigations.

## Supporting information

S1 Fig Diagram of animal protocol timeline. (TIF)

S2 Fig Log of hepatic triglyceride levels and effects of sex and diet. A. Log of hepatic triglyceride levels and effects of sex. This diagram illustrates the significant difference obtained from the ANOVA analysis between females and males (p = 0.016). Males on a HF diet had a higher hepatic triglyceride content than females on a HF diet (p = 0.026). Values shown are means ± SD. B. Log of hepatic triglyceride levels and effects of diet. This diagram illustrates the significant difference obtained from the ANOVA between mice on a LF diet and a HF diet (p = 0.0001). Males on a HF diet had higher triglycerides than females on a HF diet (p = 0.026). Values shown are means ± SD.
(TIF)

S3 Fig Representative 200x photographs of trichrome-stained liver tissue sections from each treatment group. (TIF)

S4 Fig Representative 200x photographs of CD68+ macrophages in liver sections from mice in each treatment group. (TIF)

S5 Fig Representative 200x photographs of CD3+ T-cells in liver sections from mice in each treatment group. (TIF)

S6 Fig Representative 200x photographs of Ly6+ neutrophils in liver sections from mice in each treatment group. (TIF)

S7 Fig Number of macrophages and effects of treatment and sex. A. This diagram illustrates the significant difference obtained from the ANOVA analysis between 4NQO-treated females and 4NQO-treated males (mean ± SD, p = 0.0114). B. This diagram illustrates the significant difference obtained from the ANOVA analysis between females and males (mean ± SD, p = 0.0005).
(TIF)

S8 Fig Number of T cells and effects of sex. This diagram demonstrates the significant difference obtained from the ANOVA analysis between mean values for females and males (mean ± SD, p<0.0001).
(TIF)

S9 Fig Serum ALT levels from mice in each treatment group (mean ± SD). (TIF)

S1 Table Macronutrient composition of animal diets. (PDF)

S2 Table Micronutrient composition of animal diets. (PDF)
