# Supplementary figures and images for: Fat and exposure to 4-nitroquinoline-1-oxide causes histologic and inflammatory changes in murine livers
Source: PLoS One. 2022 May 31;17(5):e0268891. doi: 10.1371/journal.pone.0268891 (PMC9154184; doi:10.1371/journal.pone.0268891)

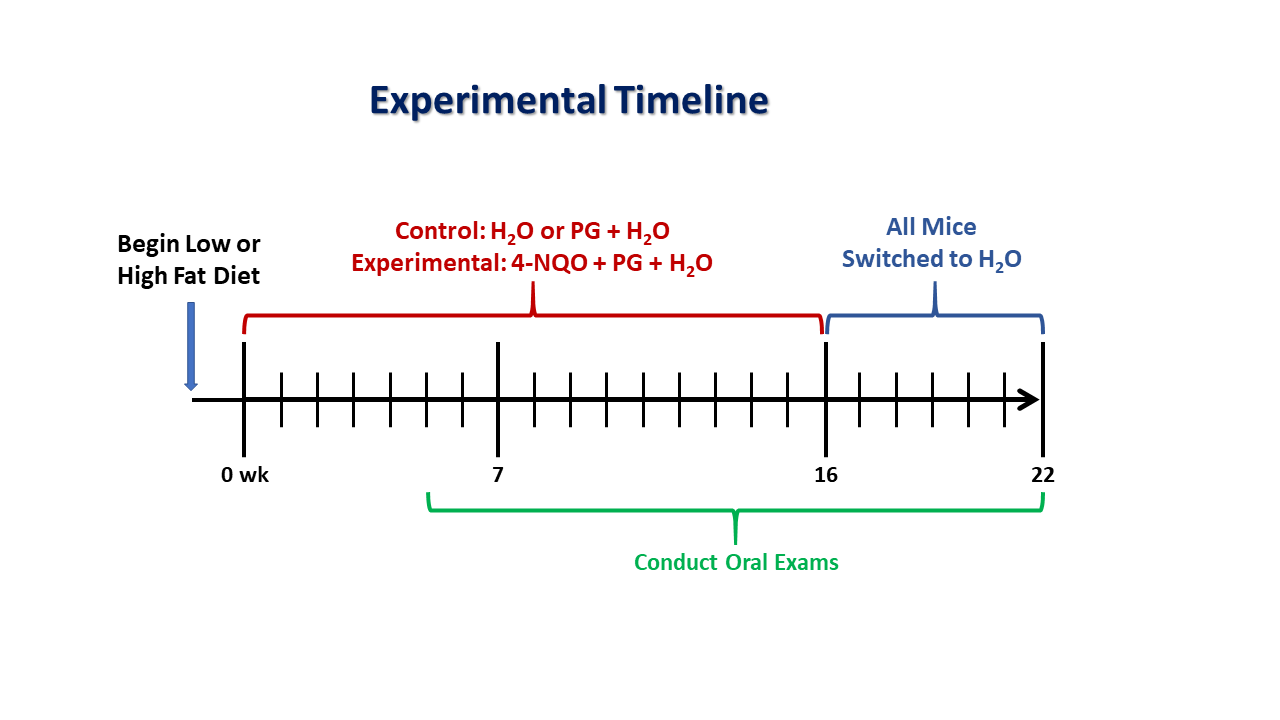

Supplement: S1 Fig — (TIF) [file pone.0268891.s001.tif]

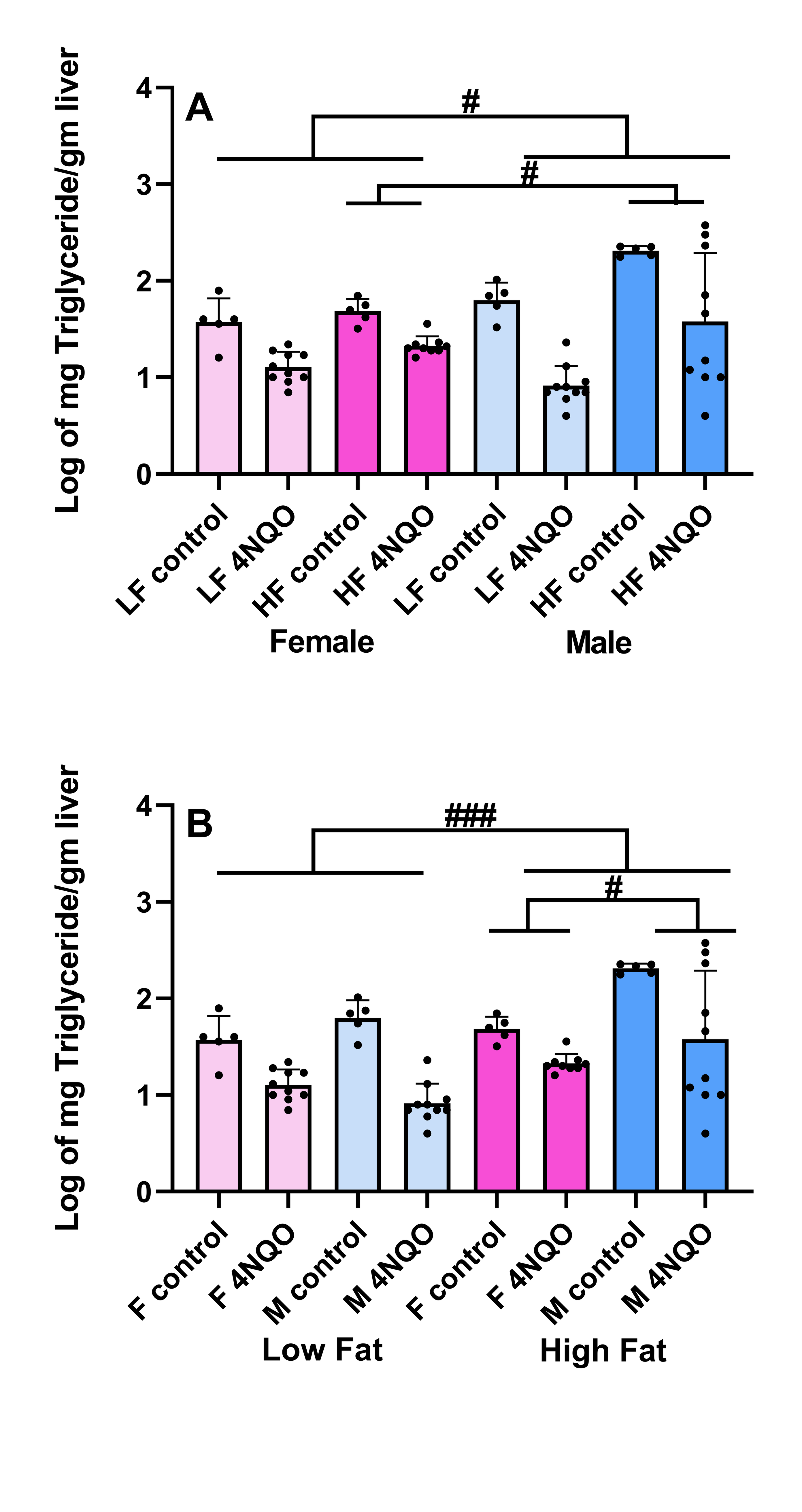

Supplement: S2 Fig — A. Log of hepatic triglyceride levels and effects of sex. This diagram illustrates the significant difference obtained from the ANOVA analysis between females and males (p = 0.016). Males on a HF diet had a higher hepatic triglyceride content than females on a HF diet (p = 0.026). Values shown are means ± SD. B. Log of hepatic triglyceride levels and effects of diet. This diagram illustrates the significant difference obtained from the ANOVA between mice on a LF diet and a HF diet (p = 0.0001). Males on a HF diet had higher triglycerides than females on a HF diet (p = 0.026). Values shown are means ± SD. (TIF) [file pone.0268891.s002.tif]

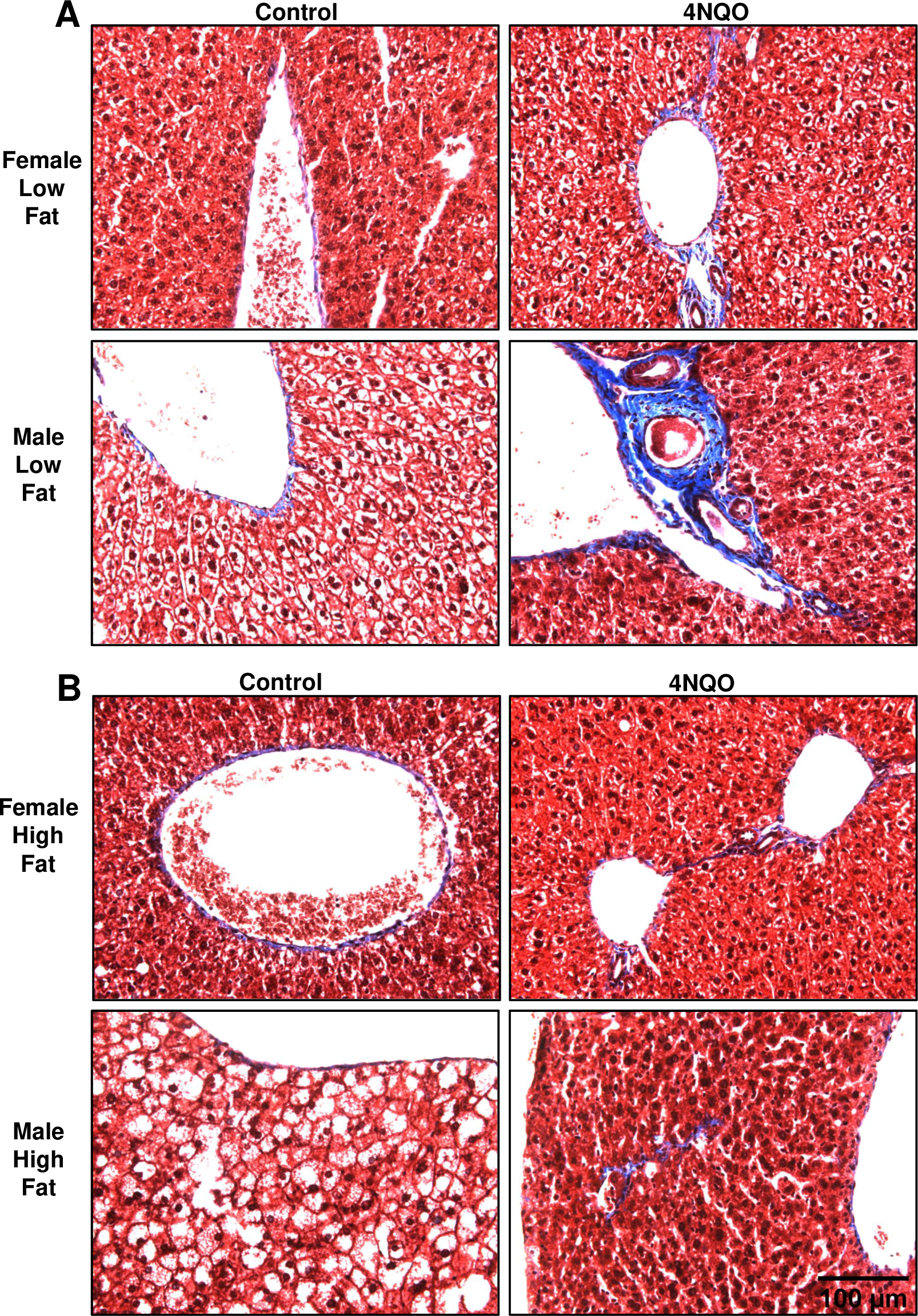

Supplement: S3 Fig — (TIF) [file pone.0268891.s003.tif]

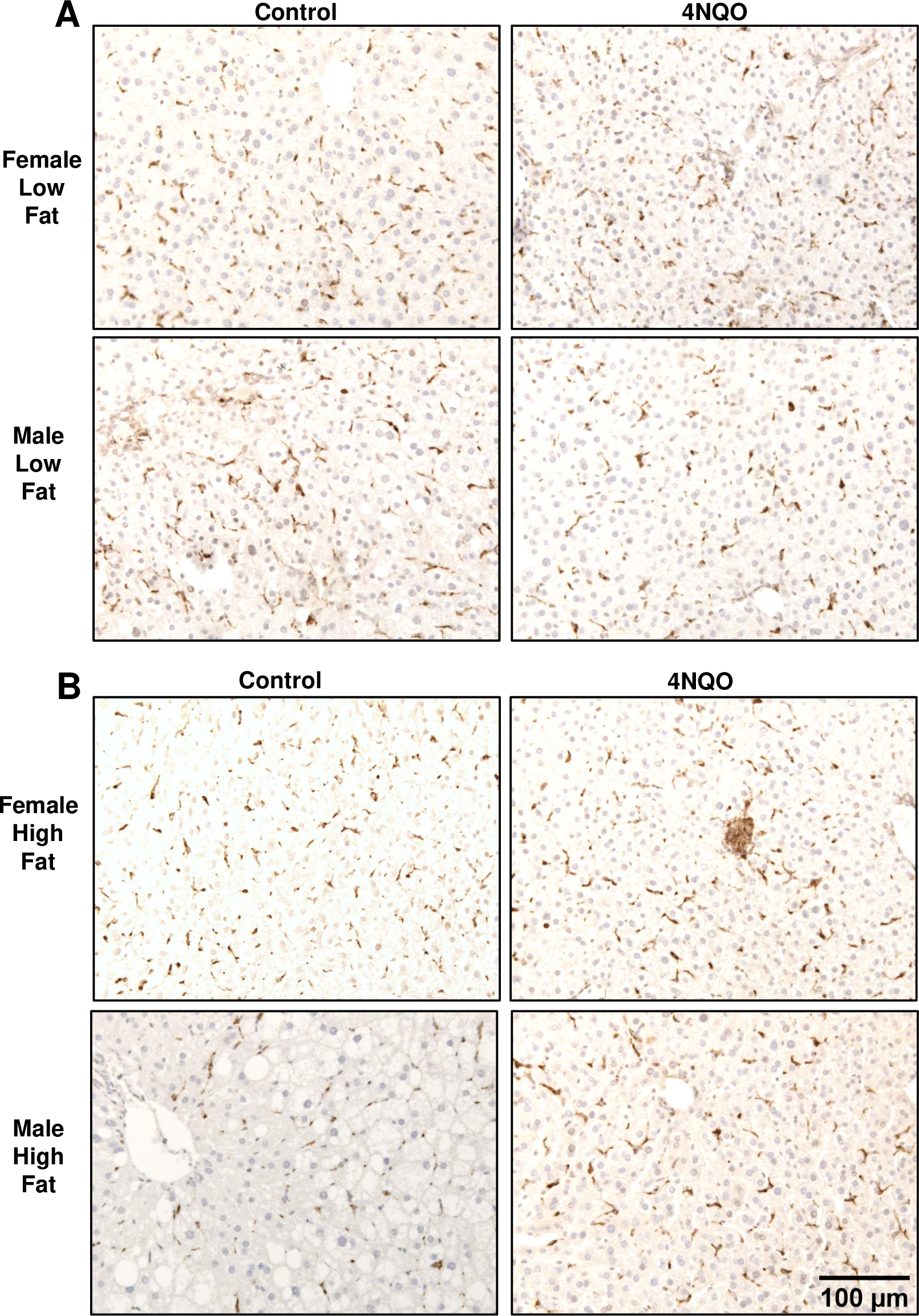

Supplement: S4 Fig — (TIF) [file pone.0268891.s004.tif]

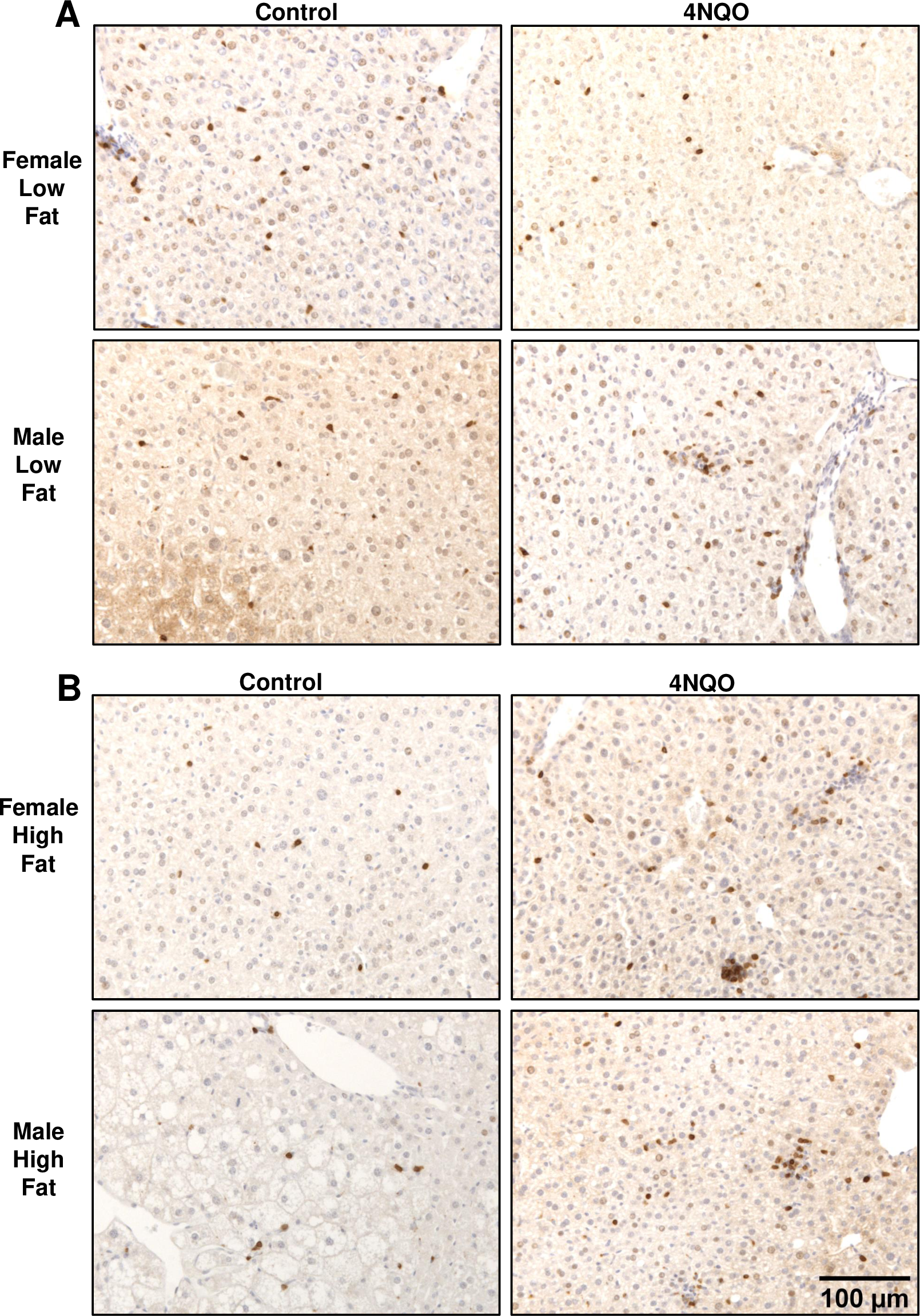

Supplement: S5 Fig — (TIF) [file pone.0268891.s005.tif]

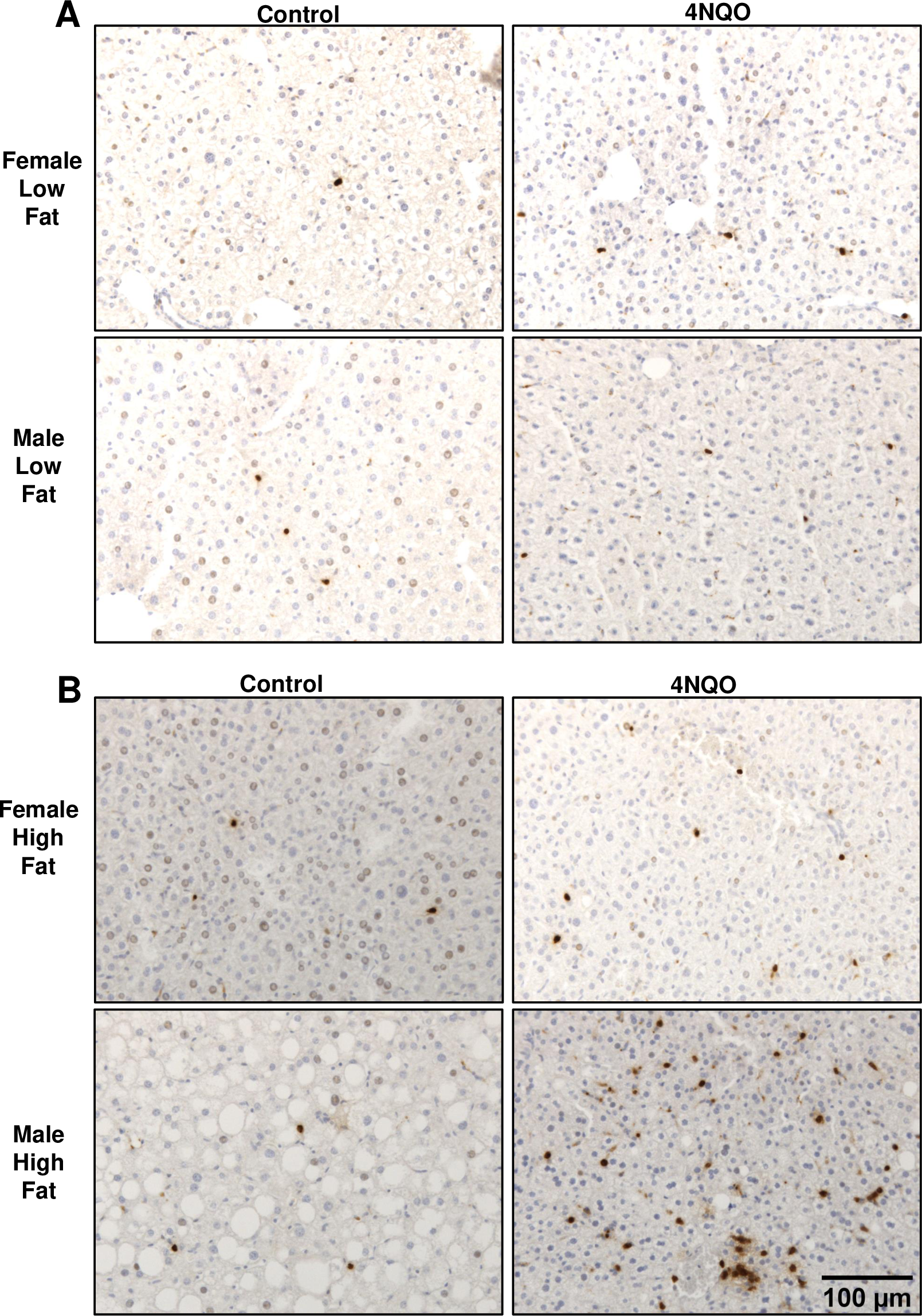

Supplement: S6 Fig — (TIF) [file pone.0268891.s006.tif]

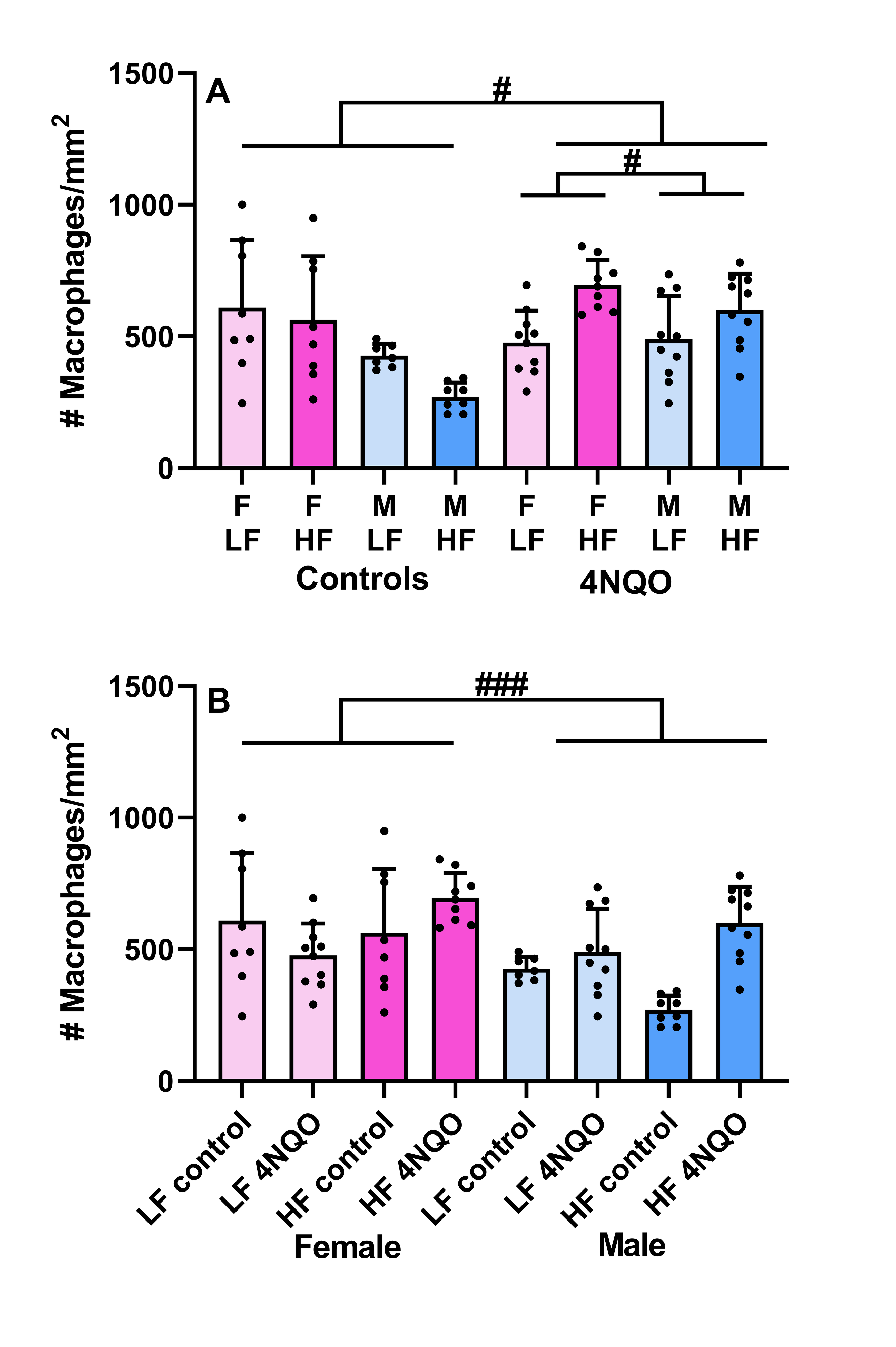

Supplement: S7 Fig — A. This diagram illustrates the significant difference obtained from the ANOVA analysis between 4NQO-treated females and 4NQO-treated males (mean ± SD, p = 0.0114). B. This diagram illustrates the significant difference obtained from the ANOVA analysis between females and males (mean ± SD, p = 0.0005). (TIF) [file pone.0268891.s007.tif]

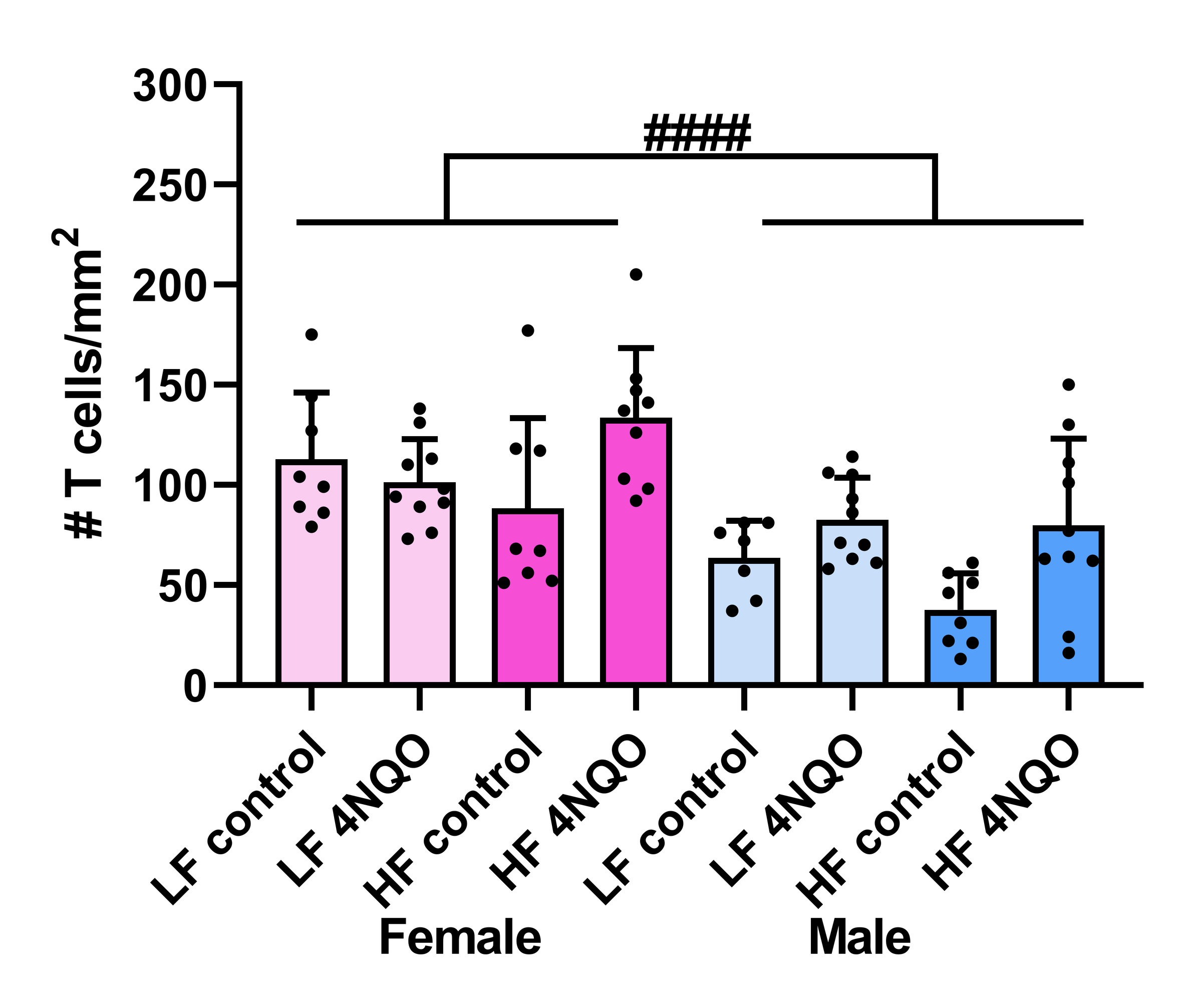

Supplement: S8 Fig — This diagram demonstrates the significant difference obtained from the ANOVA analysis between mean values for females and males (mean ± SD, p<0.0001). (TIF) [file pone.0268891.s008.tif]

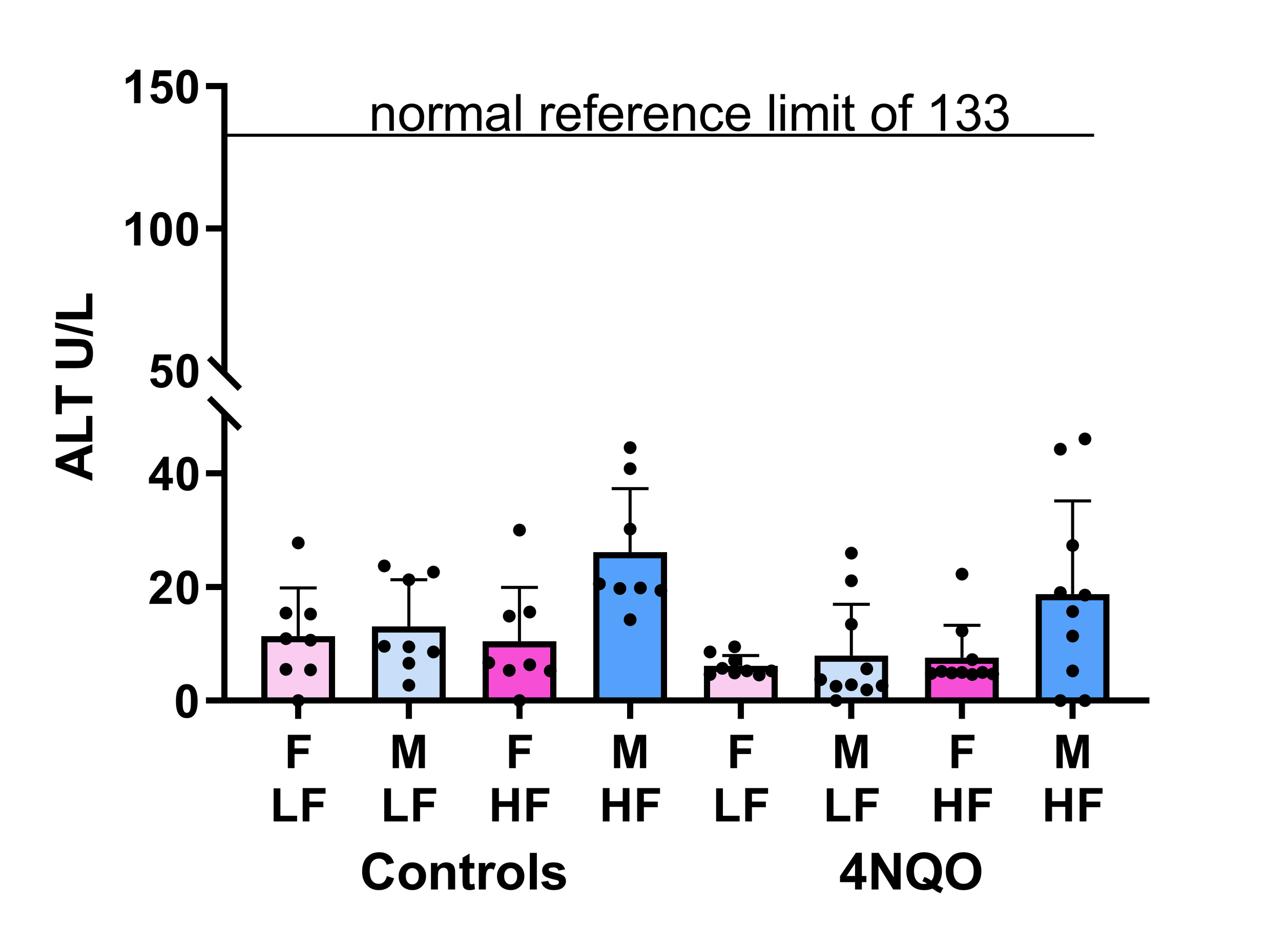

Supplement: S9 Fig — (TIF) [file pone.0268891.s009.tif]
