# Supplementary material for: Fat and exposure to 4-nitroquinoline-1-oxide causes histologic and inflammatory changes in murine livers
Source: PLoS One. 2022 May 31;17(5):e0268891. doi: 10.1371/journal.pone.0268891 (PMC9154184; doi:10.1371/journal.pone.0268891)
Supplement: S1 Table — (PDF) [file pone.0268891.s010.pdf]

Table S1. Macronutrient Composition of Animal Diets.

		Low Fat Diet D12450B: 10 kcal % fat; 35% Sucrose	High Fat Diet D12492 60 kcal% fat
Class Description	Ingredients	Grams	Grams
**Protein**	Casein, Lactic, 30 Mesh	200.00	200.00
	Cystine, L	3.00	3.00
**Carbohydrate**	Sucrose, Fine Granulated	354.00	72.80
	Starch, Corn	315.00	-
	Lodex 10	35.00	125.00
**Fiber**	Solka Floc, FCC200	50.00	50.00
**Fat**	Soybean Oil, USP	25.00	25.00
	Lard	20.00	245.00
**Mineral**	S10026B	50.00	50.00
**Vitamin**	Choline Bitartrate	2.00	2.00
	V10001C	1.00	1.00
**Dye**	Dye, Yellow FD&C #5, Alum. Lake 35-42%	0.05	0.05
	**Total:**	**1055.05**	**773.85**
