# Supplementary material for: Fat and exposure to 4-nitroquinoline-1-oxide causes histologic and inflammatory changes in murine livers
Source: PLoS One. 2022 May 31;17(5):e0268891. doi: 10.1371/journal.pone.0268891 (PMC9154184; doi:10.1371/journal.pone.0268891)
Supplement: S2 Table — (PDF) [file pone.0268891.s011.pdf]

Table S2. Micronutrient Composition of Dietary Supplements.

Mineral Supplement S10026B		
Class Description	Ingredients	Grams
Carbohydrate	Sucrose, Fine Granulated	179.82
Mineral	Potassium Citrate, Monohydrate	330.00
	Calcium Phosphate, Dibasic	260.00
	Calcium Carbonate, Light, USP	110.00
	Sodium Chloride	51.80
	Magnesium Sulfate, Heptahydrate	51.52
	Magnesium Oxide, Heavy, DC USP	8.38
	Ferric Citrate	4.20
	Manganese Carbonate Hydrate	2.45
	Zinc Carbonate	1.12
	Chromium Potassium Sulfate	0.39
	Copper Carbonate	0.21
	Ammonium Molybdate Tetrahydrate	0.06
	Sodium Fluoride	0.04
	Sodium Selenite	0.01
	Potassium Iodate	0.01
	**Total:**	**1000.00**
**Vitamin Supplement V10001C**		
Class Description	Ingredients	Grams
Carbohydrate	Sucrose, Fine Granulated	78.42
Vitamin	Vitamin E Acetate, 50%	10.00
	Niacin (a.k.a. B3)	3.00
	Biotin, 1%	2.00
	Pantothenic Acid, d, Calcium (a.k.a. B5)	1.60
	Vitamin D3, 100,000 IU/gm	1.00
	Vitamin B12, 0.1% Mannitol	1.00
	Vitamin A Acetate, 500,000 IU/gm	0.80
	Pyridoxine HCl (a.k.a. B6)	0.70
	Riboflavin (a.k.a. B2)	0.60
	Thiamine HCl (a.k.a. B1)	0.60
	Folic Acid	0.20
	Menadione Sodium Bisulfite	0.08
	**Total:**	**100.00**
